# Plasma-Treated Water Affects *Listeria monocytogenes* Vitality and Biofilm Structure

**DOI:** 10.3389/fmicb.2021.652481

**Published:** 2021-04-28

**Authors:** Oliver Handorf, Viktoria Isabella Pauker, Thomas Weihe, Jan Schäfer, Eric Freund, Uta Schnabel, Sander Bekeschus, Katharina Riedel, Jörg Ehlbeck

**Affiliations:** ^1^Leibniz Institute for Plasma Science and Technology (INP), Greifswald, Germany; ^2^Institute of Microbiology, University of Greifswald, Greifswald, Germany; ^3^School of Food Science and Environmental Health, College of Sciences and Health, Technological University, Dublin, Ireland

**Keywords:** antimicrobial, cold plasma, food production industry, MidiPLexc, PTW, sustainability, viability

## Abstract

**Background:** Plasma-generated compounds (PGCs) such as plasma-processed air (PPA) or plasma-treated water (PTW) offer an increasingly important alternative for the control of microorganisms in hard-to-reach areas found in several industrial applications including the food industry. To this end, we studied the antimicrobial capacity of PTW on the vitality and biofilm formation of *Listeria monocytogenes*, a common foodborne pathogen.

**Results:** Using a microwave plasma (MidiPLexc), 10 ml of deionized water was treated for 100, 300, and 900 s (pre-treatment time), after which the bacterial biofilm was exposed to the PTW for 1, 3, and 5 min (post-treatment time) for each pre-treatment time, separately. Colony-forming units (CFU) were significantly reduced by 4.7 log_10_ ± 0.29 log_10_, as well as the metabolic activity decreased by 47.9 ± 9.47% and the cell vitality by 69.5 ± 2.1%, compared to the control biofilms. LIVE/DEAD staining and fluorescence microscopy showed a positive correlation between treatment and incubation times, as well as reduction in vitality. Atomic force microscopy (AFM) indicated changes in the structure quality of the bacterial biofilm.

**Conclusion:** These results indicate a promising antimicrobial impact of plasma-treated water on *Listeria monocytogenes*, which may lead to more targeted applications of plasma decontamination in the food industry in the future.

## Introduction

The application of plasma-generated compounds (PGC) such as plasma-treated water (PTW) and plasma-processed air (PPA) is an emerging field of research and development (Andrasch et al., 2017; Schnabel et al., 2018, 2019a). Especially the food industry reports frequent problems with contamination of food products or its processing facilities. Often the entry of microorganisms occurs via the food itself. Fruits or vegetables, but also meat and dairy products are exposed to a natural microbial load from their environment (Heyndrickx, 2011; Matthews et al., 2014). Given the tremendous variety of ready-to-eat or ready-to-cook products in supermarkets, this problem has become increasingly important (Rodgers, 2003). A further problem is raw food that, due to their natural contamination with microorganisms, introduce new pathogens, which lead to colonization of the production facilities like drains and water connections and to contamination of the wash water and processing equipment (Knight et al., 1988; Nelson, 1990). Especially the use of PTW as wash water of the fresh-cut products, as well as the facilities, is of increasing importance for the industry in terms of environmental sustainability and thus cost-effectiveness (Rowan et al., 2007; Selma et al., 2008; Gil et al., 2009, 2015; Van Haute et al., 2013; Banach et al., 2015; Cullen et al., 2018; Patange et al., 2019b).

Instead of tap water, the plasma system could be installed upstream in the production chain and continuously produce PTW that is filled into the washing nozzles, which cleans the fresh products such as salad from heavy soiling and at the same time reduces the natural germ load. In contrast, PPA could be used for drying effects after washing or could be used directly for drying effects such as smoking fish or producing dried fruits. This development shows that the trend is moving away from direct plasma treatment of products and leads to the use of PGCs. In addition to the advantage that water is already used in the food industry and could easily be replaced by PTW, PGCs additionally offer other advantages like an easy storage and a high transportability (Schnabel et al., 2019b).

At the early 1960s the first publications concerning *Listeria monocytogenes* (*L. monocytogenes*) and the disease listeriosis were published (Seeliger, 1961; Gray and Killinger, 1966). Recently, a growing number of studies on the pathogenicity of *L. monocytogenes* have been published, which underlines its increasing importance in human and animal health care (Dhama et al., 2013; Ferreira et al., 2014; Ricci et al., 2018). *L. monocytogenes* is a facultative intracellular bacterium that can cause central nervous system (CNS) infections (Vazquez-Boland et al., 2001; Drevets and Bronze, 2008; Disson and Lecuit, 2012; Maury et al., 2017). However, it has been shown that *L. monocytogenes* is ten times more effective in invading the CNS than common pathogens such as *Streptococcus pneumoniae* or Group B Streptococci (Schuchat et al., 1997). Besides CNS infection and febrile gastroenteritis (Dalton et al., 1997; O’Toole et al., 2000; Disson and Lecuit, 2012), sepsis is one of the most common diseases caused by *L. monocytogenes* in immunosuppressed patients (Büla et al., 1995; Lorber, 1997; Opperman and Bamford, 2018; Vela et al., 2020). Less frequent are diseases like endocarditis, peritonitis, and focal infections (Doganay, 2003). In addition, *L. monocytogenes* is capable of causing materno-fetal infections, which can lead to miscarriages or infected neonates with sequelae (Fanos and Dall’Agnola, 1999; Schmidt et al., 2006; Gaini et al., 2015).

Nowadays, the Gram-positive, rod-shaped bacterium is widely known for its food-borne infection pathways (Bintsis, 2017; Luth et al., 2018). For decades, several outbreaks of food-borne listeriosis were frequently reported in North America, Europe, and in a few Asian countries (Wehr, 1987; Dalton et al., 1997; Makino et al., 2005). *L. monocytogenes* is already introduced to slaughterhouses and other food processing plants, and because of its psychrophilic character, it is often found on refrigerated food products like meat, milk, and fish (Wieczorek and Osek, 2017; Arslan and Baytur, 2019; Hanson et al., 2019). Interestingly, *L. monocytogenes* contaminations, especially of meat and dairy products, ranked only 8th among the most common disease outbreaks in the United States, following pathogens such as different *Salmonella serotypes*, *Clostridium perfringens*, *Campylobacter jejuni*, *Escherichia coli*, *Bacillus cereus*, *Staphylococcus aureus* and *Vibrio parahaemolyticus* (CDC, 2017). However, the extent of *L. monocytogenes* infections is devastating. Statistics showed that every infection with *L. monocytogenes* lead to hospitalization (CDC, 2017; RKI, 2018). If immunocompromised patients were affected, *L. monocytogenes* infections were often lethal.

*L. monocytogenes* is able to grow in multi-species biofilms. However, studies have shown that there is often competition among pathogens in multi-species biofilms and *L. monocytogenes* has much lower cell counts in the total population than in monospecies biofilms (Jeong and Frank, 1994). However, the pathogen in the multi-species biofilm showed a higher resistance to disinfectants and chemicals than in monospecies biofilms (Norwood and Gilmour, 2000).

The successful colonization of food processing plants by *L. monocytogenes* is enabled by its ability to adhere to all common surfaces occurring in food industry on which it can form biofilms (Møretrø and Langsrud, 2004). After successful adhesion, biofilms continue to grow and form sufficient protection mechanisms against disinfection like Chlorine dioxide (ClO_2_) and mechanical cleaning, which are commonly used in the food processing industry (Blackman and Frank, 1996; Korany et al., 2018; Hua et al., 2019). Conventional methods of food decontamination for fresh food include peracitic acid, lactic acid, chlorination and treatment with diluted CLO_2_ or ozone. Chlorination of food is already banned in most European Union (EU) countries due to human health hazards, but is still used in The United States or Brazil (Nguyenthe and Carlin, 1994; Baur et al., 2004; Rico et al., 2007; Jose and Vanetti, 2012). The use of diluted CLO_2_ is permitted in the EU under well-defined regulations in the industry. Due to the risk of infection from fresh food, new innovative methods are increasingly being researched, which are more efficient and less problematic than previous methods, but at the same time simple and cost-effective in its use and do not cause significant alterations to the product itself (Misra et al., 2011). Since the efficiency of the washing and sanitation processes are directly related to the microbiological quality of the product, companies are interested in the continuous optimization of these processes (Ramos et al., 2013; Do Rosario et al., 2017). Investigations showed that plasma or PGCs do not cause any changes in the texture and color of the food and still cause a significant reduction in the bacterial contamination (Vleugels et al., 2005; Xu et al., 2016; Go et al., 2019). Because many plasma sources nowadays can be operated with ambient air and tap water as a treatment source for food, the process is cost efficient and ecological. The MidiPLexc used in this work fulfills these requirements for a plasma source and could be used for industrial purposes by upscaling (Schnabel et al., 2019a).

The establishment of new processes in food processing facilities, such as PTW-dependent sterilization of food products like fresh-cut vegetables or meat, is a time-consuming and difficult process (Schnabel et al., 2019a, 2020). For this reason, it is even more important to show the effect of PTW against food-dependent hosted pathogens. Therefore, this work provides new insights about the antibacterial effect of microwave-induced PTW against *L. monocytogenes* biofilms. Therefore, the aim of this study is to visualize the impact of PTW treatment by the MidiPLexc on *L. monocytogenes* biofilms in terms of killing the cells to generate a comprehensive model of the antimicrobial spectrum of PTW generated by microwave induced plasma sources.

## Materials and Methods

### Plasma Source

**FIGURE 1:**
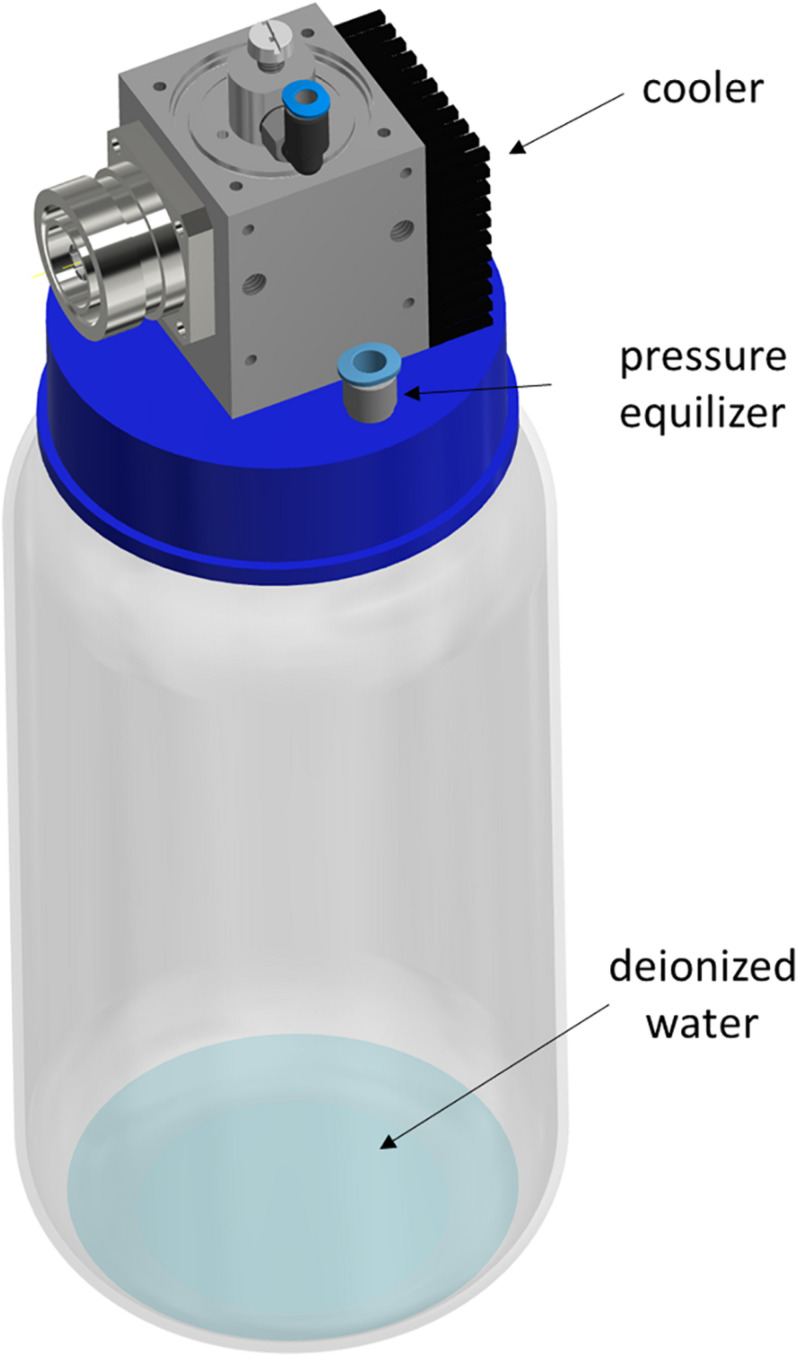
Schematic illustration of the MidiPLexc. The image shows the MidiPLexc with a 1 l glass bottle connected to the bottle adapter below the plasma source as a 3D model. Image is modified according to Handorf et al. (2020a).

The MidiPLexc (Handorf et al., 2020a) is a microwave-driven plasma source and an extension of the MiniMIP (Baeva et al., 2012; Figure 1). In contrast to the MiniMIP, this plasma source can be operated with compressed air instead of argon gas, which leads to lower operation costs. In addition, it is possible to treat different amounts of liquids with the microwave-induced plasma gas, because of its integrated bottle adapter (Handorf et al., 2020a, b). The bottle was filled with water and connected to the operating plasma source. The effluent of the MidiPLexc extends directly into the bottle and the formed PPA sinks down and converts the water into PTW (Figure 1). This PTW is ready to use and can be transported to the place of application. The MidiPLexc was operated with compressed air as working gas and a gas flow of 1 slm with a forward power of 80 W and a reverse power of 20 W. After 30 min running time a stable gas flow could be maintained and the effluent was ejected steadily and continuously out of the plasma source.

### Generation of the PTW by the MidiPLexc

A 1 l glass bottle was filled with 10 ml of deionized water (DW) and integrated into the bottle adapter of the MidiPLexc for the production of the PTW. In our experiments, we used the terms pre- and post-treatment times. The pre-treatment defines the contact time of the DW with the plasma gas and the post-treatment is equivalent to the contact time of the PTW with the biofilm. For the biological investigations, three different pre-treatment times (100, 300, and 900 s) and three different post-treatment times (1, 3, and 5 min) for each pre-treatment time were used. Each post-treatment time for the biofilms was performed in triplicates per experimental day. Each test was performed in four biological replicates. This yielded in *n* = 12 for each post-treatment time.

### Bacterial Strain and Growth Conditions

*L. monocytogenes* (ATCC 15313) was used for cultivation because of its well-known ability to form biofilms (Borges et al., 2012; Vazquez-Sanchez et al., 2017). In the beginning, 1 l Brain Heart Infusion (BHI) broth (Roth, Karlsruhe, Germany) was prepared, autoclaved, and its pH value was adjusted to pH 6 by adding 10 M hydrochloric acid (HCl). This was adapted according to the results of a previous study (Buchanan and Bagi, 1999). Additionally, the BHI medium was pumped through a 0.2 μm polyether sulfone (PES) filter system (VWR, Darmstadt, Germany) using a vacuum pump and was sterile filtrated because of possible recontamination during the pH adaption. A colony was removed from an inoculated agar plate using a 10 μl inoculation loop, which has been suspended in 50 ml BHI medium afterward and was incubated for 24 h at 30°C without shaking. On the next day, 1 ml of the suspension was adjusted to an optical density (OD) at 600 nm of 0.100 by using 10 mm diameter polystyrene cuvettes in a UV-3100PC Spectrophotometer (VWR). This suspension was used for biofilm cultivation by pipetting 300 μl per well in a 96-well plate and incubated again for 24 h at 30°C without shaking to ensure cell adhesion. Afterward, the medium was removed to discard the non-adhered cells, and 300 μl of fresh medium was added. After another 24 h of incubation at 30°C in the dark without shaking, the PTW treatment was started.

### PTW, ClO_2_, Ethanol, and HCl Treatment of *L. monocytogenes* Biofilms

After careful removal of the medium, 300 μl PTW produced by the MidiPLexc for the different post-treatment times were added to the biofilms. The same procedure was done for the investigations with ClO_2_, HCl and ethanol. As the different chemicals have their own pH value and to avoid that the different pH values have an influence on the antimicrobial properties of the chemicals, their pH values were adjusted to the pH value of the PTW to eliminate the pH effect. For these experiments, the pH values were adjusted with 10 M HCl added to the ClO_2_ and ethanol solution until the required pH value of 1.271 was reached. For the HCl solution, 10 M NaOH solution was used to adjust the pH value. In order to exclude a manipulation of the results by the intrinsic absorption of ClO_2_, a blank value of the 15 ppm ClO_2_ solution before and after addition of the (2,3-Bis-(2-Methoxy-4-Nitro-5-Sulfophenyl)-2H-Tetrazolium-5-Carboxanilid) (XTT) solution was measured photometrically and the absorption values were subtracted from those of the samples.

Only one post-treatment time was performed at the same time to avoid any drying effects on the biofilms. Afterward, the solution was removed, and the biofilm was mechanically detached from the surface by the shear forces of the liquid through pipetting, before being dissolved in 300 μl phosphate buffered saline (PBS) (pH 7.2; according to Sörensen). To ensure the transfer of the entire biofilm, this step was repeated two times in total, which resulted in a final suspension volume of 600 μl. This suspension was used for colony-forming units (CFU) assay (2.5), fluorescence assay (2.6), as well as XTT assay (2.7). The mechanical detachment of the biofilms was omitted for fluorescence microscopy (2.8), confocal laser scanning microscopy (CLSM) (2.9), and atomic force microscopy (AFM) (2.10).

### Determination of the Remaining CFU After PTW Treatment

To determine the CFU after PTW treatment of the biofilm, 100 μl were taken from the 600 μl suspension (2.3), and a serial dilution was performed. This was done by diluting the specimen in a ratio of 1:10 with maximum recovery diluent (MRD; 0.85% NaCl, 1% tryptone). The controls were finally diluted 1:1,000,000 and the samples 1:1,000. Each dilution step was plated on BHI agar by pipetting 10 μl per dilution onto the plate and spread out by using the tilting technique. The plates were incubated for 24 h at 30°C, without shaking. The colonies for the respective dilution levels were counted manually, and the CFU/ml values were calculated by using the formula:

$$C\,F\,U=\frac{10^{x}}{v}*\frac{\sum c_{y}+\sum c_{y+1}}{n_{y}+0,1\,n_{y+1}}$$

where 10^x^ is the dilution factor for the lowest dilution, v is the volume of diluted cell suspension per plate in ml, Σc_y_ is the total number of colonies on all (n_y_) plates of the lowest evaluated dilution level, 10^–x^, and Σc_y__+__1_ is the total number of colonies on all (n_y__+__1_) plates of the next-highest dilution level evaluated, 10^–(x+1)^. The calculation is explained in more detail in Bast (2001) and Handorf et al. (2019).

**FIGURE 2:**
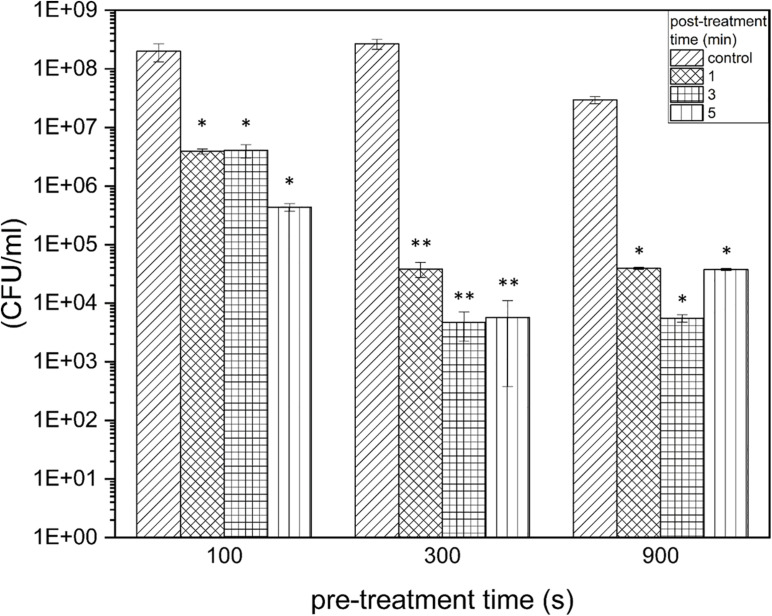
Colony-formingun its of *Listeria monocytogenes* biofilms after PTW treatment. The graph shows the colony forming units of *Listeria monocytogenes* biofilms before and after plasma treatment. The data are presented in the form of a grouped bar chart where the values on the *x*-axis represent the different pre-treatment times and the different bars represent the various post-treatment times. The experiment was performed in four independent experiments with three technical replicates each. *p ≤ 0.05 and **p ≤0.005, tested with ANOVA. All treated groups were testes in relation to the control group.

The propagation of error was calculated for each treatment group. This finally resulted in four different error propagations for each treatment time, from which the weighted error was calculated and used as error bars in the illustration (Figure 2; Gränicher, 1994). The experiments were repeated fourfold with *n* = 3, which finally resulted in *n* = 12. Additionally, the reductions were expressed as reduction factors (RF) resulting from the subtraction of the logarithmic value of the treated biofilms from the logarithmic value of the control biofilms.

### Fluorescence LIVE/DEAD Assay

The LIVE/DEAD BacLight Bacterial Kit (Thermo Scientific, Waltham, United States) was used to detect membrane damage of the bacteria caused by the plasma treatment and was prepared according to the product description. For 300 μl sample, 0.9 μl of the applied dye was added to each well and the 96-well plate was incubated for 15 min at room temperature in the dark on a rotary shaker at 80 rpm. After the incubation time, the 96-well plate was read with a microplate reader (Varioskan-Flash, Thermo Scientific) at an excitation wavelength of 470 nm and an emission wavelength of 530 nm for SYTO9 and 630 nm for propidium iodide (PI). Finally, the ratio Green/Red cells (G/R) was calculated by dividing the two fluorescence values obtained from one sample. The calculated values of the samples were visualized as a percentage compared to the control biofilm values and displayed using Origin 2019b software (OriginLab, Northampton, United States). The experiments were performed on four independent experiments with *n* = 3 per sample. This corresponds to a total number of *n* = 12 per test combination.

### Fluorescence Microscopy

For fluorescence microscopy, the biofilms were cultivated and observed in special transparent 96-well plates with glass bottom (Eppendorf, Hamburg, Germany). These plates are tissue-culture (TC)-treated like the polystyrene plates for the other tests to ensure an equal surface for cell attachment. However, the glass bottom offers a lower noise ratio, because of this we changed to plates with glass bottom for the microscopy. Therefore, they were stained with the LIVE/DEAD assay as it is mentioned in (2.6) after plasma treatment, but without dissolving the sample in 300 μl PBS. After staining, the fluorescent solution was carefully removed from the biofilm and the biofilms were microscopically examined with the Operetta CLS high content imaging device (PerkinElmer, Hamburg, Germany). Four fields of view were brought together digitally for the total biofilm images. A 5x objective (air, *NA* = 0.16, Zeiss, Oberkochen, Germany) was used. SYTO9 was excited by a 475 nm (110 mW) LED, and the fluorescence was collected with a 525 ± 25 nm bandpass filter. PI was excited by a 550 nm (170 mW) LED, and the emission light was collected through a 610 ± 40 nm bandpass filter. Laser autofocus (785 nm) provided exact focusing across all fields of view. Three stacks were merged into a maximum intensity projection in order to display topographic features in the z-plane (focus ± 25 μm). Thirty stacks with a distance of 1.5 μm between each stack were assembled to 3D biofilms using a 40x air objective (*NA* = 0.6). Three-dimensional reconstruction, image stitching, and quantification were done using Harmony 4.8 software (PerkinElmer).

### CLSM

For CLSM, the biofilms were treated as mentioned before under (2.3, 2.4, and 2.6). After staining the biofilms and removing the supernatant, the samples were examined with the Zeiss LSM 510 microscope (Carl Zeiss, Jena, Germany) with a 63x objective (water, *NA* = 0.1). For the detection of SYTO9, an argon laser was used and excited at 488 nm and emitted at 505–530 nm using a band pass filter. PI was also excited at 488 nm but emitted at 650 nm using a long pass filter. The 3D images of the biofilms were displayed with the ZEN 2009 software (Carl Zeiss) on an area of 100 × 100 μm with z-stacks at a distance of 5 μm.

### XTT Assay

The XTT assay visualizes cell vitality after plasma treatment by detecting the metabolic activity of the cells and thus their redox potential via trans-plasma membrane electron transport. The activation solvent of the XTT solution contains N-methyl dibenzopyrazine methylsulfate (PMS) as an intermediate electron carrier and was mixed with the XTT solution in a ratio of 1:50. This activated solution was brought together with the sample in a 1:3 ratio and incubated for 20–24 h on a horizontal shaker at 80 rpm and 37°C in the dark. On the next day, the 96-well plate was measured at 470 nm with the Varioskan Flash device. In addition to the samples, the blank value, which consists the activated XTT solution without sample, was measured in threefold at 670 nm for each experiment and the mean value of the three blank values was subtracted from the sample values. The experiments were performed on four independent experiments with *n* = 3 per sample. This corresponds to a total number of *n* = 12 per test combination.

### Atomic-Force Microscopy

Because the well plates cannot be measured directly with the AFM, the biofilms were grown on sterile polyethylene terephthalate, glycol-modified (PET-G) cover slips (13 mm, Sarstedt, Nümbrecht, Germany). They were grown on 50 ml Gelrite (Duchefa, Haarlem, Netherlands), which was pipetted into the 12-well plate directly after autoclaving for curing. In each well, 1 ml Gelrite was given on whose surface the Cover slips were applied. However, the biofilms were cultured as described in 2.3 and treated as mentioned in 2.4, whereby the corresponding volumes were adapted to the size of the plate and thus 1 ml BHI medium per well was added. For the AFM recordings only the 5 min post-treatment times of the different pre-treatment times were examined in addition to the controls due to the complexity of the measurements. To avoid dehydration of the samples, they were stored in humidity chambers for the duration of the experiment. The AFM measurements were carried out on a DI CP II SPM (Veeco, Plainview, United States), which was mounted on a vibration-free object table (TS 150, TableStable, Zwillikon, Switzerland). The setup was standing on an optical bench encased by an additional acoustic protection. The AFM was equipped with a linearized piezo scanner, on which the coverslips were mounted on a metal sample holder with leading tabs. The samples were measured using cantilevers (Bruker, Karlsruhe, Germany) with nominal spring constants of *k* = 0.1–0.6 N/m in contact mode. The pictures were taken by a scanning speed of 0.4 Hz by a picture size of 20 μm^2^ and a set point = 8 N/m. Pictures were edited with Gwyddion (Czech Metrology Institute, Brno, Czechia).

### Scanning Electron Microscopy (SEM)

The biofilms were cultivated in 12-well plates as described under 2.10 and plasma generation was performed as described under 2.2. All biofilms were treated for 5 min with the PTW of the different pre-treatment times. The control biofilm was treated with PBS for the same post-treatment time. After treatment, the biofilms grown on the cover slips were transferred to small plastic dishes and placed in a desiccator. In the desiccator, the biofilms were dried for 24 h at 1 mbar. The next day, the biofilms were removed from the desiccator and prepared for SEM examination. The biofilm samples were placed in a vacuum chamber for 24 h and then coated with a thin electrically conductive layer of gold in a sputtering coater SCD 050 (Bal-Tec, Switzerland). The coating of the sample made it possible to eliminate the electrical charging of the sample in a scanning electron microscope (SEM) in order to suppress artifacts in SEM micrographs. Samples prepared in this way were analyzed in SEM Jeol JSM7500F microscope. The SEM was operated in a gentle beam mode: accelerating voltage 2 kV, working distance 3 mm, and magnification 10–50 k.

## Results

### Optimal Treatment Window of Pre-treatment and Post-treatment Times Concerning the Reduced Proliferation of Biofilm Cells

In terms of an optimal treatment window of *L. monocytogenes* biofilms, the effect on the proliferation ability of the pathogen was investigated, and it was demonstrated at which settings a significant reduction in the proliferation ability could be achieved. The proliferation assay showed a reduction of 1.95 × 10^8^ cells (RF of 1.71, *p* = 0.0067) already after 100 s pre-treatment time, 1 min post-treatment time. This is on the rise with increasing post-treatment times (RF of 2.66 after 5 min, *p* = 0.0066). With a longer pre-treatment time, even a stronger reduction could be observed, which corresponds to an RF of 3.84 (300 s, 1 min, *p* = 0.0022), as well as a RF of 3.73 (900 s, 3 min, *p* = 0.0207; Figure 2). With a pre-treatment time of 300 s and longer, the highest reductions were achieved with a post-treatment time of 3 min and slightly lower reductions were achieved with longer post-treatment times (5 min) (Figure 2). In terms of total cell counts, the strongest influence on cell proliferation was measured after 300 s, 3 min PTW treatment. In total, a maximum reduction of *L. monocytogenes* biofilm cells from 2.67 × 10^8^ to 4.68 × 10^3^ (reduction of 4.7 log_10_, *p* = 0.0022) was achieved.

### PTW Treatment Leads to a Strong Reduction in the Vitality of Biofilm Cells

**FIGURE 3:**
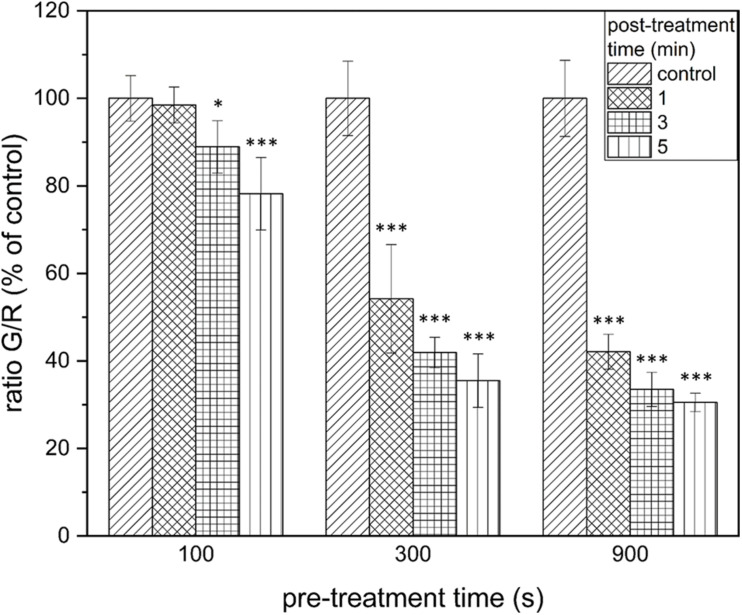
LIVE/DEAD assay of *Listeria monocytogenes* biofilms after PTW treatment. The figure shows the LIVE/DEAD assay of *Listeria monocytogenes* biofilms after treatment with plasma-treated water. The different bars show the control biofilms and the different post-treatment times and the *x*-axis shows the different pre-treatment times of the water. The experiment was performed in four independent experiments with three technical replicates each. *p ≤ 0.05 and ***p ≤ 0.0005, tested with ANOVA. All treated groups were testes in relation to the control group.

The results of the LIVE/DEAD assay provide informations about membrane damages of the cells and thus their vitality. The results of the vitality assay at a pre-treatment time of 100 s showed that a significant reduction of vitality by 25.9 ± 11.5% (*p* = 0.5 × 10^–5^) could only be achieved after 5 min post-treatment time (Figure 3). With a pre-treatment time of 300 s, a reduction by 45.8 ± 12.4% (*p* = 1.07 × 10^–6^) could be achieved after 1 min post-treatment time. This increased progressively to 58.1 ± 3.5% (300 s, 3 min, *p* = 9.26 × 10^–10^), and 64.5 ± 6.1% (300 s, 5 min, *p* = 5.25 × 10^–9^; Figure 3, center). The increased reduction could also be measured at 900 s pre-treatment time. In this case, the strongest measured reduction in vitality by 69.5 ± 2.1% (*p* = 2.43 × 10^–10^) occurred after 5 min post-treatment time (Figure 3, right).

### The Reduction of the Metabolism of Biofilm Cells Was Only Detectable After Long Pre-treatment and Post-treatment Times

**FIGURE 4:**
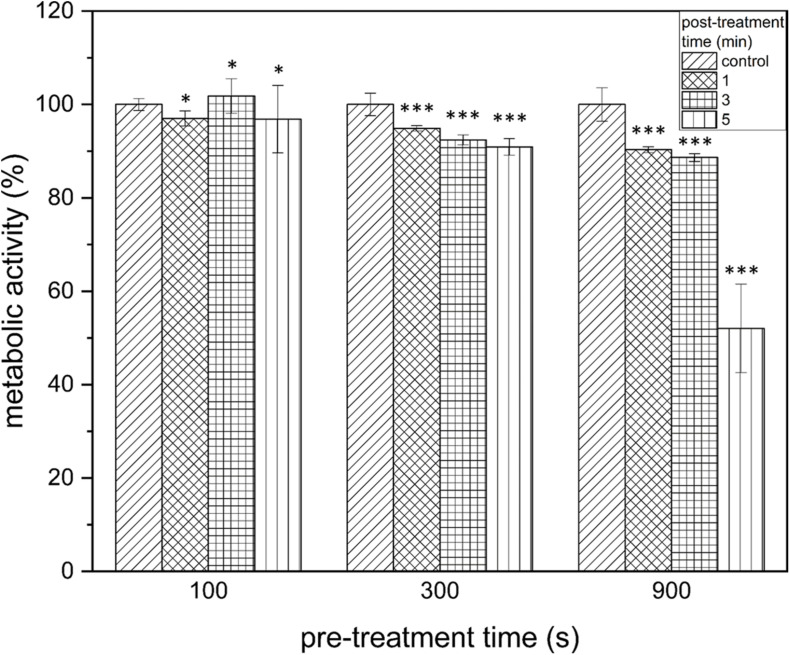
XTT assay of *Listeria monocytogenes* biofilms after PTW treatment. The influence on the metabolic activity of the biofilms after treatment with plasma treated water is shown in this image. The grouped bar chart shows the different post-treatment times and the *x*-axis shows the different pre-treatment times of the biofilms. The experiment was performed in four independent experiments with three technical replicates each. *p ≤ 0.05 and ***p ≤ 0.0005, tested with ANOVA. All treated groups were testes in relation to the control group.

By measuring the reduction of the metabolic activity of the biofilm cells after PTW treatment with the XTT assay, no significant changes could be observed at 100 s pre-treatment time despite the length of the post-treatment time (Figure 4, left). If PTW was used, which was treated with the MidiPLexc for 300 s, only very low reductions with a maximum of 9.1 ± 1.77% (300 s, 5 min, *p* = 4.276 × 10^–14^) could be detected in the various post-treatment times (Figure 4, center). At 900 s pre-treatment time, comparable reductions of 11.4 ± 0.89% (900 s, 3 min, *p* = 1.041 × 10^–8^) could be detected. However, a significantly higher reduction of 47.95 ± 9.47% (*p* = 1.073 × 10^–5^) could be demonstrated at 900 s pre-treatment and 5 min post-treatment time, where the dynamic effect of the metabolism of the cells began (Figure 4, right).

### The Antimicrobial Characteristics of Substances Frequently Used in the Food Industry Are Partially pH Value Specific

**FIGURE 5:**
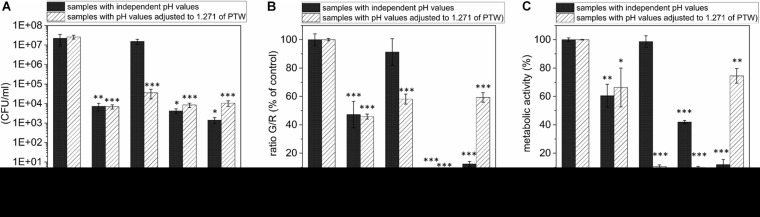
Comparison of the effects of PTW, ClO_2_, 10 M HCl, and 70% Alcohol against *L. monocytogenes* biofilms investigating CFU, Fluorescence and XTT assay and influence of the pH value concerning these effects. **(A)** CFU assay of *L. monocytogenes* biofilms after treatment with different chemicals frequently used in the food industry with their own pH value after preparation and compared with the results of the chemicals adapted to the pH value of the PTW of 1.271. **(B)** Fluorescence assay of *L. monocytogenes* biofilms **(C)** XTT assay of *L. monocytogenes* biofilms. *p ≤ 0.05, **p ≤ 0.005, and ***p ≤ 0.0005, tested with ANOVA. All treated groups were testes in relation to the control group.

In order to give an idea of the extent to which the effect of PTW is comparable to conventional industrial disinfectants and substances used to remove biofilms, it was compared with these substances concerning the proliferation ability, vitality and metabolic activity of the cells after treatment. The treatment of DW for 900 s led to a pH value of 1.27. To investigate whether the antimicrobial effect of PTW compared to 15 ppm (15 mg/l) ClO_2_ was based on the different pH values of these solutions, the antimicrobial effect of these substances in their original pH value were compared with the antimicrobial effect of the pH values adapted to the pH value of the PTW with respect to CFU (Figure 5A), fluorescence (Figure 5B), and XTT assay (Figure 5C). The CFU count showed a strong pH dependence for ClO_2_ and a slight dependence for 10 M HCl (Figure 5A). These effects were confirmed in the fluorescence assay. Here, a strong pH dependence could also be observed for ClO_2_ (Figure 5B). However, a strong dependence for 10 M HCl also becomes apparent in this case. Interestingly, the effect on cell metabolism for all substances is extremely pH dependent. In this case, a clear effect could also be observed with 70% alcohol (Figure 5C). ClO_2_ in its used concentration (15 ppm) has a pH value of 4.288, which means that the pH value was lowered by 3.017 in the experiment. This showed that ClO_2_ only unfolds its antimicrobial effect in very low pH ranges. Nevertheless, the antimicrobial effect of ClO_2_ could exceed that of PTW only in the XTT assay at low pH values (Figure 5C). Here a reduction of the metabolism by 89% (*p* = 1.873 × 10^–8^) was achieved compared to 33% (*p* = 0.013) by PTW. With 10 M HCl, the effect was antagonistic. Here the pH value of the 10 M HCl, which was –1.609 in the used concentration, was increased by a pH value of 2.88. This has only a slightly weaker effect on the proliferation of the cells with 10 M HCl, but a very strongly weakened effect on the vitality and metabolism of the cells.

### Fluorescence Microscopy Revealed Inactivation Kinetics From the Top to the Bottom of the Biofilm

**FIGURE 6:**
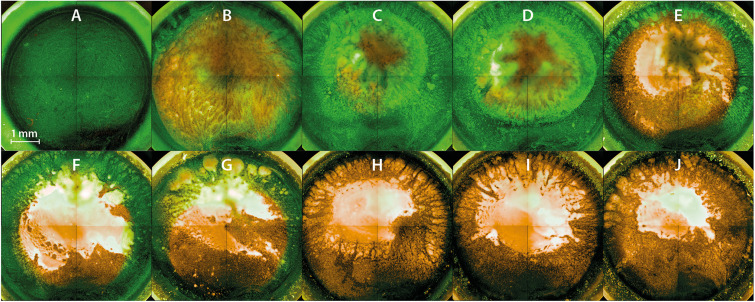
Fluorescence microscopy of *Listeria monocytogenes* biofilms after PTW treatment. The images show inverse footage of the *Listeria monocytogenes* biofilms. The pre-treatment time defines the time period in which the water came into contact with the plasma gas. The post-treatment time is the period of time where the PTW came into contact with the biofilm. The biofilms are stained with SYTO9 (green) for living cells and propidium iodide (PI) (red) for dead cells. **(A)** Control biofilm **(B)** 100 s pre-treatment, 1 min post-treatment **(C)** 100 s pre-treatment time, 3 min post-treatment time **(D)** 100 s pre-treatment, 5 min post-treatment **(E)** 300 s pre-treatment, 1 min post-treatment **(F)** 300 s pre-treatment, 3 min post-treatment **(G)** 300 s pre-treatment, 5 min post-treatment **(H)** 900 s pre-treatment, 1 min post-treatment **(I)** 900 s pre-treatment, 3 min post-treatment **(J)** 900 s pre-treatment, 5 min post-treatment.

Fluorescence microscopy should give a visual impression of the effects of PTW treatment against the biofilms. All fluorescence microscopic images were inverse footages of the biofilms. The cells of the untreated biofilm showed only vital (green fluorescence) cells (Figure 6A). The 3D images of the same footage confirmed this and showed a very dense biofilm with mushroom-shaped ridges within the biofilm extending to a height of about 60 μm, whereas the average biofilm was limited to a thickness of about 20 μm. This area was characterized by a strong staining, which resulted in a very dense structure compared to the mushroom-shaped areas (Supplementary Figure 1). The biofilms showed progressively more inactivated cells using 900 s pre-treated PTW (Figures 6B–J). The 3D image of this biofilm showed a nearly complete dead biofilm (Supplementary Figure 2). Only a few very small hotspots of living cells were visible in the biofilm. The dynamics comparing the different treatment times showed that the inactivation started in the center and expanded to the outside (Figures 6B–E).

### CLSM Showed a Partial Inactivation of Biofilms Predominantly in the Central Area

**FIGURE 7:**
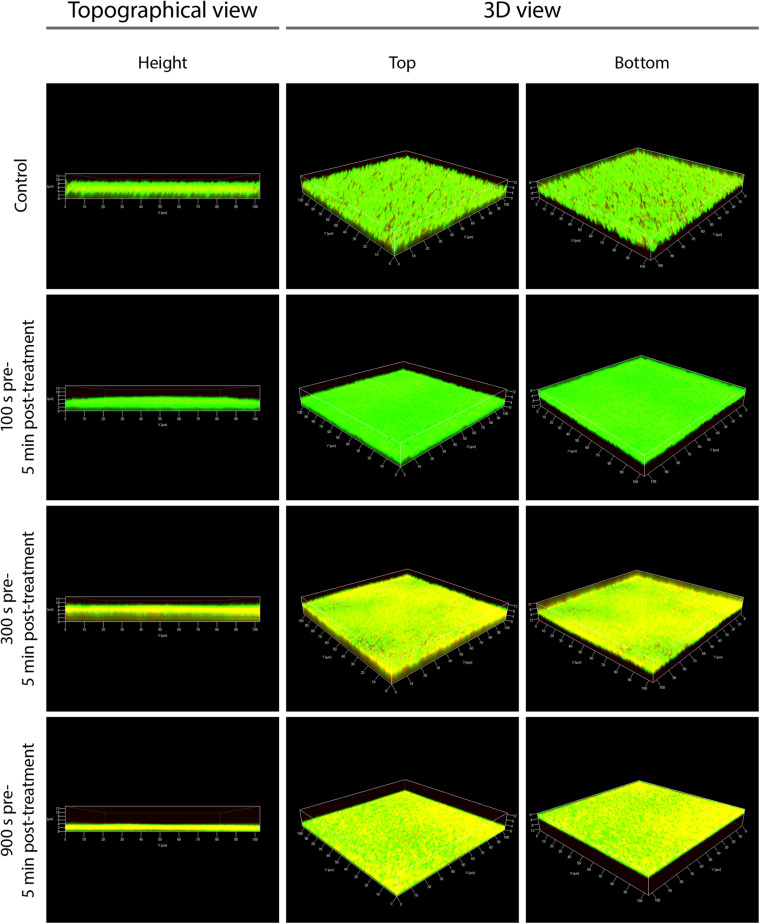
Confocal laser scanning microscopy (CLSM) of *Listeria monocytogenes* biofilms after PTW treatment. The picture shows the untreated control biofilm and the *Listeria monocytogenes* biofilms processed with plasma-treated water in confocal laser scanning microscopy (CLSM). **Left panels** show a topographical view of the biofilm layer (height view of the biofilms in μm). Central and **right panels** show 3D images with a top and a bottom view of the biofilms, respectively. The pre-treatment time is the processing time of the water by the plasma source (MidiPLexc) and the post-treatment time is the contact time of the PTW with the biofilm. For each biofilm, an area of 100 × 100 μm is visualized.

In contrast to fluorescence microscopy, CLSM 3D models could be used to display images of the entire biofilm and not just specific areas. This gave a good overview of the holistic effects of PTW treatment. The control biofilms showed continuous living cells and a relaxed structure (Figure 7, topographical view). After 100 s pre-, 5 min post-treatment time, no changes in the LIVE/DEAD staining of the biofilm cells could be detected. Only living cells were visible in the biofilm, but the biofilm appeared much denser compared to the control biofilm (Figure 7, see 100 s pre-, 5 min post-treatment). After 300 s pre-, 5 min post-treatment time, no changes in the thickness of the biofilms compared to the control biofilm could be detected. The biofilms were about 10 μm thick as well as the control biofilms. However, mainly cells in the central area of the biofilm were stained yellow, which indicates inactivation of the cells but no death (Figure 7, see 300 s pre-, 5 min post-treatment). After 900 s pre-, 5 min post-treatment, there was, no change in the staining of the cells visible compared to the 300 s pre-treatment. However, there was a significant removal of biofilm mass of about 6 μm, resulting in a biofilm thickness of 4 μm (Figure 7, see 900 s pre-, 5 min post-treatment). The biofilm matrix have appeared flat with a homogenous structure.

### AFM Images Showed Alterations in the Plasticity of the Biofilm Surface

**FIGURE 8:**
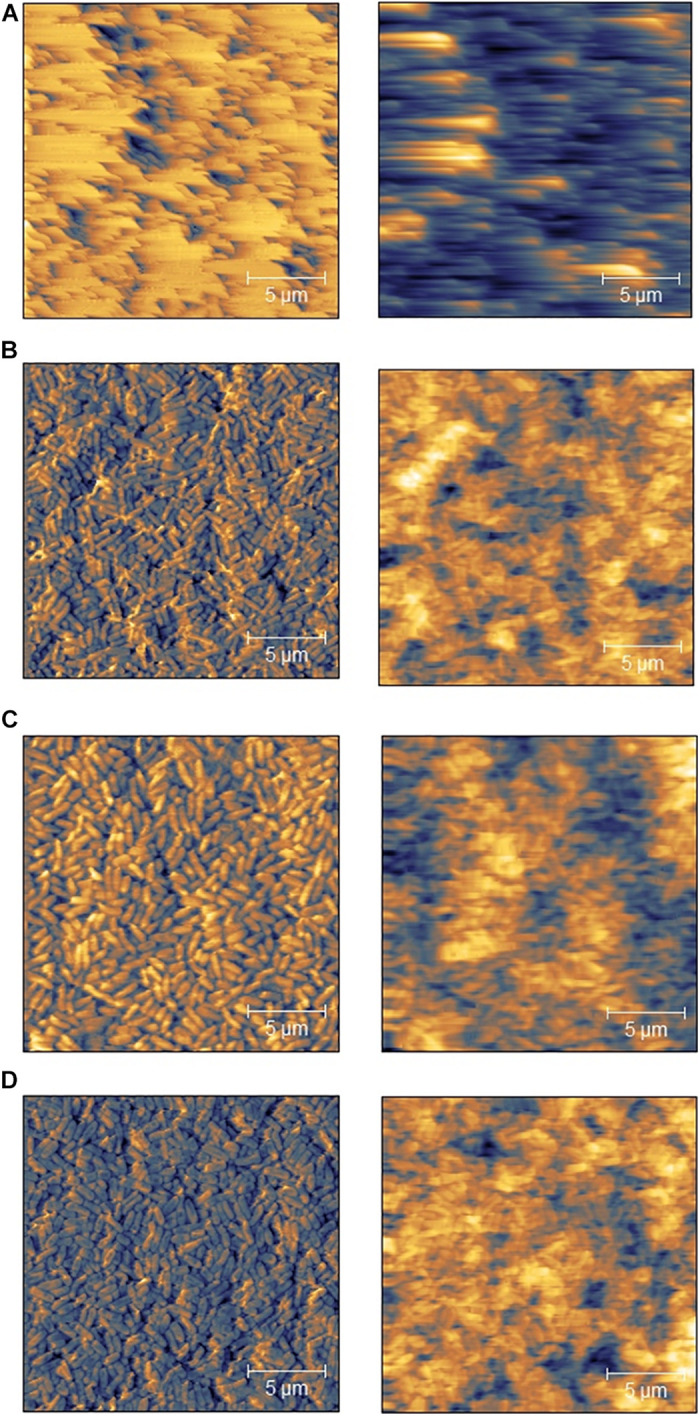
Atomic-force microscopy (AFM) of *Listeria monocytogenes* biofilms after PTW treatment. AFM images of *Listeria monocytogenes* biofilms. Left) topographical images right) error images. The pre-treatment time defines the period in which the water were exposed to the plasma gas. The post-treatment time represents the period, where the PTW were exposed to the biofilm. **(A)** Control biofilm **(B)** 100 s pre-treatment, 5 min post-treatment **(C)** 300 s pre-treatment, 5 min post-treatment **(D)** 900 s pre-treatment, 5 min post-treatment.

With the help of AFM images, conclusions could be drawn from the PTW treatment on the surface condition of the biofilms. The AFM images of the control biofilms showed long blurred structures of the biofilm surface, which allowed conclusions about the softness of the surface (Figure 8A). These long error lines indicate that the surface is very soft and the needle of the cantilever cannot reproduce it correctly and pushes the actual contour as it moves. This results in such long lines (Figure 8A). In contrast, the treated biofilms could be displayed very well defined and structured (Figure 8B). Concerning the cell morphology, there was hardly any change visible after treatment. However, comparing the biofilm surface structure of increasing treatment times led to the conclusion that the cells appeared significantly larger and somehow swollen (Figures 8B–D).

### SEM Confirmed Membrane Damages of the Biofilm Cells After Plasma Treatment

**FIGURE 9:**
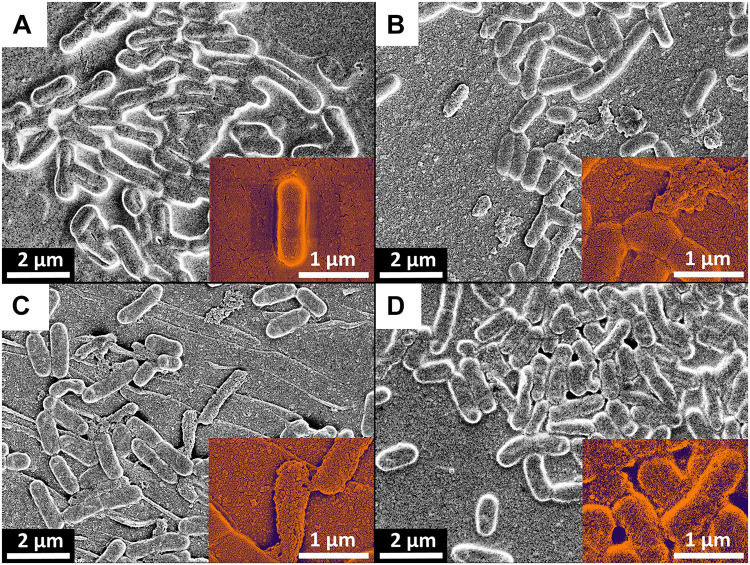
Scanning-electron microscopy (SEM) of *Listeria monocytogenes* biofilms after PTW treatment. SEM images of *Listeria monocytogenes* biofilms. **(A)** Control biofilm **(B)** 100 s pre-treatment, 5 min post-treatment **(C)** 300 s pre-treatment, 5 min post-treatment **(D)** 900 s pre-treatment, 5 min post-treatment. The red arrows indicated cells with membrane damages.

SEM images demonstrated cell alterations by the PTW treatment (Figure 9). Morphological analysis showed that the bacteria surface of the control sample is compact and full (Figure 9A). In contrast to this, treated bacteria samples suggest a flat morphology and some of them are completely crushed, as shown by detailed sections at 50 k magnification (Figures 9B–D). All samples show the nanostructured texture of the gold layer and short cracks in the surface, which are related to the coating process in vacuum (Figure 9).

## Discussion

Key findings:

• The most important factor in relation to the antimicrobial effect of PTW is the treatment time of the water with the plasma source and the associated concentration with reactive species.
• The antimicrobial effect against *L. monocytogenes* is relatively low. This contrasts with the already known antimicrobial effect of this PTW against P. fluorescens.
• Comprehensive investigation of antimicrobial activity using multiple biological assays such as CFU, fluorescence and XTT is crucial to draw conclusions about the antimicrobial effect.
• Reactive nitrogen species play a crucial role in the antimicrobial activity of the microwave-induced plasma source MidiPLexc.
• Plasma treatment leads to membrane damage of the biofilm cells.

The detailed mechanisms of the PGC effects remain unclear, but recent studies revealed a coherence of the antimicrobial potential with the concentration of reactive oxygen (ROS) and nitrogen (RNS) species (Handorf et al., 2020a, b). The microwave-induced MidiPLexc plasma source provides a temperature regime that favors the production of RNS such as nitrate (NO_3_^–^), nitrite (NO_2_^–^), and nitrogen monoxide (NO). Beside these dominating compounds, peroxynitrite (ONOO^–^) and hydrogen peroxide (H_2_O_2_) has been generated in various quantities and may be considered as intermediates for more stable RNS or ROS, respectively (Handorf et al., 2020a).

Generally, the acidic character of the PTW increases its antimicrobial effect (Oehmigen et al., 2010), by contributing to chemical redox reactions within the PTW (Ikawa et al., 2010). For instance, the reaction of ONOO^–^ to the peroxonitric acid (HNO_4_) seems to be a pivotal factor. This is, followed by the reactions of nitrous acid (HNO_2_) and nitric acid (HNO_3_), which derive from NO_2_^–^ and NO_3_^–^, respectively, and seem to be responsible for the antimicrobial effect of PTW.

An influence of the temperature can be excluded (Handorf et al., 2020a). Even direct biofilm treatments with the MiniMIP, the previous version of the MidiPLexc, revealed no temperature influences on the antimicrobial behavior against biofilms (Handorf et al., 2019). Additionally, the temperature increases by approximately 3.5°C only during the production of PTW with the MidiPLexc (Handorf et al., 2020a). Consequently, the antimicrobial effect of a PTW solution is achieved by the reactive oxygen/nitrogen species (RONS).

The investigation of the effects of PTW in the field of decontamination of food and surfaces in food production is still in its infancy. While there have been numerous studies investigating the effects of plasma gas on food products (Frohling et al., 2012; Rod et al., 2012; Schnabel et al., 2015a, 2018; Tappi et al., 2016), surfaces (Li et al., 2012; Scholtz et al., 2015; Govaert et al., 2019), and packaging (Pankaj et al., 2014; Schnabel et al., 2015b), less has been reported about the treatment with PTW (Jung et al., 2015; Xu et al., 2016; Thirumdas et al., 2018; Yong et al., 2018). The treatment of *L. monocytogenes* monospecies and multispecies biofilms with *Pseudomonas fluorescens* on lettuce with plasma gas resulted in reductions to undetectable levels after 60 s treatment time for the monospecies biofilms and a reduction of 2.2 log_10_ steps for *L. monocytogenes* in a multispecies biofilm with *P. fluorescens* (Patange et al., 2019a). It has been shown that multispecies biofilms of *L. monocytogenes* showed a higher resistance against plasma gas than monospecies biofilms. In our work, we demonstrated that *L. monocytogenes* biofilms have a much higher resistance against PTW than other bacteria like *P. fluorescens* (Handorf et al., 2020b).

The results of CFU, fluorescence and XTT assay clearly show that the pre-treatment time and thus the concentration of PTW with reactive species is of more crucial importance than the post-treatment time and thus the reaction time of PTW with the biofilm. This also indicates that the antimicrobial effect of PTW occurs relatively rapid. Comparing the results of the fluorescence assay with those of the XTT assay, the result shows that the cells of the biofilm suffer membrane damage but still have an intact metabolism. This is one of the most significant signatures that the cells were not killed but only attacked by the PTW treatment. It is known that especially Gram-positive cells with membrane damage can still survive or even reverse membrane permeabilization (Garcia et al., 2007).

In the field of antimicrobial research of plasma and PGCs, the effect of these substances on the extracellular polymeric substances (EPS) of biofilms is becoming increasingly important. However, the effect of PTW on different EPS is still not fully understood. Thus, a comparison with other studies is very difficult, since in addition to the plasma source and application method (PPA or PTW), the pathogen strain (Doijad et al., 2015), growth time and treatment time also play an important role. In addition, the growth temperature (Patange et al., 2019a) and the overgrown material or food product (Ziuzina et al., 2015) also have a decisive influence. A previous conducted and comparable study to this work showed the effect of PTW on *P. fluorescens* monospecies biofilms. It demonstrated a reduction of 6 log_10_ steps compared to a control biofilm with a CFU of 10 log_10_. Mechanistically, a clear removal of the biofilm material by the PTW treatment could be proven as well as cell death within that work (Handorf et al., 2020b). In the present study, a reduction of about 5 log_10_ steps compared to a control biofilm of 8 log_10_ was detectable (2.5). The CFU shows that higher post-treatment times sometimes show worse results than lower ones. This can be explained by the fact that the cells tend to form clusters after prolonged contact with PTW. This seems to be a defense mechanism, whereby the outer cells of the cluster protect the internal cells and thus more cells survive (Steenackers et al., 2016; Handorf et al., 2020b). However, this only protects to a limited extent, so that significantly longer treatment times also lead to cluster killing. The important difference compared with the previous study, however, lies in the vitality and metabolism results and in the microscopic analysis. For the *L. monocytogenes* biofilms, a removal of the biofilm material could also be proven, but a significantly lower effect in the LIVE/DEAD assay in combination with the 3D image of the CLSM (Supplementary Material). On the one hand, this again showed the compelling evidence of further investigations concerning the effects of PTW besides the CFU assay and, on the other hand, it rather indicates an inactivation of the cells more than cell death (2.6).

The effect of PTW compared to conventional disinfection methods such as alcohol disinfection of surfaces or ClO_2_ as a wash water additive shows that PTW could act as an innovative addition to these agents. PTW could also be used directly for the produce in the same way as diluted ClO_2_ solution (Trinetta et al., 2012), but also for surface disinfection like alcohol (Lembke, 2001). Interestingly, ClO_2_ shows hardly any microbial effect without additional acidification. Seventy percent alcohol shows a stronger effect than PTW, but cannot be used directly on the produce. The fuming hydrochloric acid served as positive control and shows that *L. monocytogenes* even at these acidic pH values partly survives better than the alkaline alcohol. This could also be an indicator for the better effectiveness of PTW against Gram-negative bacteria. Even with fuming hydrochloric acid, some of the cells were still able to proliferate, although an negligible amount.

PTW treatments can lead to changes in cell morphology and thus in the structural nature of the entire biofilm (Begum et al., 2018). This could also be proven with the results of AFM. The long error lines in the control biofilm (Figure 8A) are caused by changes in the nature of the biofilm surface. If the surface is particularly soft, these error lines can occur. The applied force of the cantilever causes the cantilever to sink into the surface and during the movement across the biofilm, the cantilever cannot reproduce the structure correctly. On the contrary, the treated biofilms are perfectly visualized, which means that the surface becomes stronger and stiffer.

The effect of plasma on the extracellular matrix can be decisive. Various studies have already demonstrated the effect of plasma on the EPS of biofilms (Srey et al., 2014; Ziuzina et al., 2014; Bourke et al., 2017; Gilmore et al., 2018). Studies have shown that different species can produce various amounts of EPS (O’Toole et al., 2000; Vu et al., 2009). Furthermore, the composition of the different EPS of the pathogens could also play an important role. *P. fluorescens* generates EPS matrix, which is rich in proteins and carbohydrates. The dominant components are proteins, which may explain the mucous nature of the biofilms in combination with polysaccharides (O’Toole et al., 2000; Simoes et al., 2003; Molobela et al., 2010). *L. monocytogenes*, strain ATCC 15313 forms a thick and compact matrix, which seems to contain much less EPS, which mainly covers the overgrown surface. The EPS of *L. monocytogenes* consists of mainly polysaccharides and teichoic acids (Colagiorgi et al., 2016). These results showing the differences in the physical composition of the biofilm and its properties and therefore, could explain their higher resistance to plasma treatment. With the help of SEM images, it could be shown that the cells were altered by the PTW treatment (Figure 9). After plasma treatment, a stronger fragmentation could be observed (Figures 9B–D). This could explain the variation between control biofilms and plasma-treated biofilms in the AFM images (Figure 8). Nevertheless, these results showed that the effect of PTW against *L. monocytogenes* biofilms justifies investigations that are more intensive.

This raises the question about the effect of plasma treatments against EPS. It is well established that EPS has a protective effect for the biofilm during plasma treatment with plasma gas or direct contact with the plasma effluent. However, the results of this work in combination with previous work showed a rather antagonistic effect (Handorf et al., 2020b). This could be due to the high water binding capacity of the biofilm (Allison, 2003; Evans, 2005; Flemming and Wingender, 2010). Macroscopically, when treating the biofilms with PTW, it is easy to see that the PTW is partly completely absorbed by the biofilm. This absorption leads to a certain dilution effect of the concentrated components of the PTW, but also to a longer contact time with the cells of the biofilm beyond the actual post-treatment time. This may explain why Gram-negative pathogens like *P. fluorescens*, which form a much thicker biofilm, are more susceptible to PTW treatment. Since it is generally accepted that the thicker the biofilm, the less frequently the cells in the lower layers of the biofilm are affected (Mah and O’Toole, 2001). Due to the high water content of the ECM, the PTW seems to overcome this effect, quickly dissolve the components of the PTW in the water of the ECM, and thus distribute them in the biofilm. This leads to the fact that the ECM is partially destroyed by the PTW treatment, which leads to the fact that the spaces between the cells of the biofilm become smaller and the biofilm mass shrinks. This phenomenon could explain why the biofilm appears denser at high PTW treatments in the CLSM images. With the high concentrations of NO_2_^–^ and NO_3_^–^, as used after the pre-treatment times applied for *L. monocytogenes* biofilm treatment, the dilution effects of the EPS play a rather minor role (Handorf et al., 2020a). With regard to the application, a further concentration of the water contained in the EPS by subsequent plasma gas treatment could have a far-reaching synergistic effect. This effect has already been demonstrated in previous work and will most likely be further enhanced by the enrichment of the PTW components in the biofilm matrix (Schnabel et al., 2019b).

This basic research offers an extension of existing knowledge on the direct treatment of food products on an industrial scale. For this purpose, lettuce samples have already been treated with PTW according to industrial standards and examined for its qualitative change in color and microbial load (Schnabel et al., 2019b, 2020, 2021).

Summarizing, this work shows the complexity of the problem of microbiological contamination in the food production industry. Different pathogens and applications require different experimental setups. Therefore, the choice of plasma source, settings and treatment times must always be adapted to the fundamental problem in the industry. The plasma source shown in this paper has the advantage that it is small, can be easily set up in different locations, and has a suitcase variant for transport. Furthermore, the produced PTW can be easily transported in bottles. Thus, the plasma source can be positioned directly in the desired area of the industrial plant. Due to the operation with compressed air, this plasma source creates a large cost reduction compared to conventional plasma sources, which normally work with noble gases such as argon as carrier gas, especially in continuous operation. The disadvantage of this plasma source is the relatively small amount of water volume, which can be further processed to PTW. More than 10 ml per treatment should not be produced with this plasma source. However, this is not directly disadvantageous, since this plasma source is only a model variant for already upscaled plasma sources, which are based on the same principle and are already used in pilot scale facilities in the industry. Thus, this study forms an additional data acquisition as a supporting database to the already used upscaled plasma sources.

## Conclusion

This work vividly demonstrates the complexity of the effect of PGCs on *L. monocytogenes* biofilms. The complex implementation of microbiological methods and their interpretation as a sophisticated system clearly showed that these investigations could only be evaluated as an all-embracing system. While single results like the CFU showed considerable reductions, the microscopic images show rather minor membrane damages of the cells. In comparison to already published studies, this work shows that a decisive factor in the antimicrobial effect of PTW is the cell wall structure of the bacteria and those Gram-positive pathogens such as *L. monocytogenes* seems to be much more resistant to PTW treatment than Gram-negative pathogens like *P. fluorescens*. This is of crucial importance for industrial applications.

## Data Availability Statement

The raw data supporting the conclusions of this article will be made available by the authors, without undue reservation.

## Author Contributions

OH, KR, and JE: conceptualization. OH, VP, TW, JS, EF, and SB: methodology. OH, VP, TW, JS, and SB: software and validation. OH: formal analysis, investigation, data curation, and writing—original draft preparation. KR and JE: resources and project administration. TW, JS, US, SB, KR, and JE: writing—review and editing. OH, VP, TW, JS, and SB: visualization. US: supervision. VP, SB, and KR: funding acquisition. All authors have read and agreed to the published version of the manuscript.

## Conflict of Interest

The authors declare that the research was conducted in the absence of any commercial or financial relationships that could be construed as a potential conflict of interest.
